# Datascape: exploring heterogeneous dataspace

**DOI:** 10.1038/s41598-024-52493-7

**Published:** 2024-04-05

**Authors:** Jakez Rolland, Ronan Boutin, Damien Eveillard, Benoit Delahaye

**Affiliations:** 1 Nantes Université, École Centrale Nantes, CNRS, LS2N, UMR 6004, 44322 Nantes, France; 2 Bio Logbook, 44200 Nantes, France

**Keywords:** Data processing, Ecological modelling, Computational biology and bioinformatics, Mathematics and computing, Computational science, Computer science, Risk factors, Biooceanography, Ecological modelling

## Abstract

Data science is a powerful field for gaining insights, comparing, and predicting behaviors from datasets. However, the diversity of methods and hypotheses needed to abstract a dataset exhibits a lack of genericity. Moreover, the shape of a dataset, which structures its contained information and uncertainties, is rarely considered. Inspired by state-of-the-art manifold learning and hull estimations algorithms, we propose a novel framework, the datascape, that leverages topology and graph theory to abstract heterogeneous datasets. Built upon the combination of a nearest neighbor graph, a set of convex hulls, and a metric distance that respects the shape of the data, the datascape allows exploration of the dataset’s underlying space. We show that the datascape can uncover underlying functions from simulated datasets, build predictive algorithms with performance close to state-of-the-art algorithms, and reveal insightful geodesic paths between points. It demonstrates versatility through ecological, medical, and simulated data use cases.

The quantity of data collected in various fields of societal impacts, such as economy, ecology, medicine, and industry, is growing daily, while the nature of the data is constantly evolving and becoming more heterogeneous^1^. Over the last decades, data has been produced only by designed scientific experiments that assure homogeneity of the data and the lack of bias. Instead, data is collected thanks to sensors, smartphones, and computers opportunistically, producing uncontrolled, heterogeneous data^2^. Datasets embed knowledge that data science aims to identify for designing decision support tools, such as those to measure the impact of anthropic activities^3,4^ or health status^5^. For this purpose, large datasets with many points and dimensions are often abstracted by objects like statistical distributions, statistical metrics, or parametric models, which qualify predictions, comparisons, and classification. For example, a linear regression model between two variables will summarize the 2-dimensional cloud of points induced by two variables^6^, a non-linear model such as stated in^7^ is adapted to certain non-linear processes.

However, whether of high practical interest, these approaches are not generic as the choice of abstraction relies foremost on the question to answer and the data to process. Indeed, statistical models need the data to meet specific requirements for use^8^. Furthermore, these state-of-the-art abstractions do not allow an exploration of the knowledge without performing tedious meta-analyses driven by hypotheses^9^.

Given the growing data heterogeneity, we advocate for a generic framework for analyzing datasets and extracting insights through their integration, facilitating comprehensive data exploration. We endorse that a generic approach to describing data should focus on the topology and geometry of the data rather than its sole statistical properties. A dataset is sampled on a multidimensional underlying space—the dataspace—with a structure and shape. Two complementary properties can describe this space: (1) its topology, i.e., which describes how elements of the space connect themselves, and (2) boundaries, i.e., the contour of the space, which describes an inside and an exterior. These combined properties allow us to consider natural data uncertainties without altering the data topology. The description of these properties relies on seminal works, including the notion of manifold (i.e., a generalization of curves and surfaces in high dimension), manifold learning algorithms^10^, and recent topological data analysis approaches^11,12^.

This study states and formalizes a framework—the datascape—to abstract a dataset thanks to a k-nearest-neighbor graph and the decomposition of the underlying data space in convex hulls. Worth noticing, this study aims not to provide a faithful visualization of a dataset like tSNE, UMAP or PHATE but to illustrate how exciting properties of the datascape can be used. As a significant benefit, the datascape can leverage the power of graph theory to measure distances between points while considering the topology of the underlying space, exhibit path, and geodesics, and assess whether a given point belongs to the dataspace. We illustrate how the datascape can be used to abstract and explore a dataset, study its shape, follow up on the evolution of a particular point, make predictions, clusters, and more. We demonstrate the performance of the datascape abstraction, which we applied to simulated, longitudinal medical, and spatial ecological data. In particular, we show that the datascape can uncover an underlying function from noisy simulated data and can be used to build predictive algorithms in the medical field with performance close to state-of-the-art predictive algorithms or to reveal insightful geodesic paths between ecological stations.

## Results

### Datascape rational

**Figure 1:**
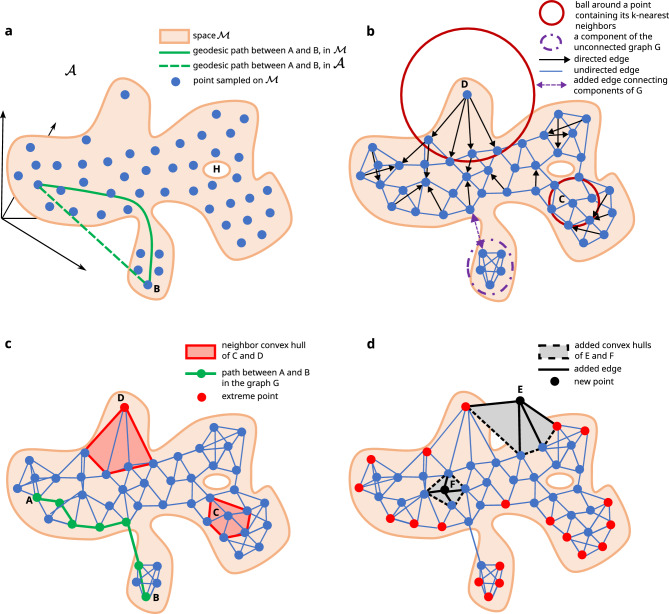
Conceptual steps to build a datascape: (**a**) sampling dataset *X* from a continuous d-manifold ${\mathcal {M}}$ with a boundary. Within the datascape framework, the distance between two points is measured along the shortest path that follows the data’s shape. (**b**) The topology of the d-manifold ${\mathcal {M}}$ is abstracted with the nearest neighbor graph *G*. The graph might contain unconnected components. In this case, edges are added to connect them. (**c**) Directed edges are converted to undirected edges to symmetric neighbor relationships. The space ${\mathcal {M}}$ is approximated by the union of the neighbor convex hulls of all points. (**d**) Extreme points located on the border of the datascape are identified and colored in red. A new point added to the datascape is considered inside the latter if it is inside any of the neighbor convex hulls (see point F). Otherwise, they are considered outside the datascape (see point E).

Whereas “Materials and methods” presents an extensive description of the datascape, this section proposes a less formal description for general and trans-application understanding sake. Let $X=\{x_i\}_{i \in {\mathbb {N}}}$ be a set of points of dimension $d \in {\mathbb {N}}$ sampled from a space ${\mathcal {M}}$ embedded in an ambient space ${\mathcal {A}}$. We aim to construct a faithful abstraction of ${\mathcal {M}}$—the datascape ${\mathcal {D}}$—using the points *X*. However, building such an abstraction is a complex task for heterogeneous data.

We identify three sources of heterogeneity in a dataset *X*. (1) Certain phenomena live in non-linear spaces, such as systems that behave under non-linear processes like population dynamics^13^, reaction–diffusion systems, and biological processes^14^. (2) Using samples or observations to build a dataset can produce a biased representation^15^. For example, rare phenomena occurrences will be less sampled than common ones. Sporadic occurrences might not even appear. Consequently, the data *X* might over-represent some parts of the underlying space ${\mathcal {M}}$. At the same time, some points might appear isolated because they lie in a region of the space ${\mathcal {M}}$ poorly represented in the sample. (3) High dimensionality of data, often called the curse of dimensionality, can also produce heterogeneity^16^ as the distances between points increase drastically. The sample offers a highly incomplete view of the actual underlying space with holes and cavities (see “Materials and methods” section for a formal definition)^17^. The higher the dimension, the bigger the dataset must be to approximate its underlying space faithfully. For being robust to this heterogeneity, an abstraction of a dataset must (1) be resilient to non-linearity, (2) minimize prior hypothesis and therefore the number of hyper-parameters it relies on, and (3) allow the description and comparison of elements of the dataset in respect of the shape of the underlying space.

Constructing the datascape relies on several steps illustrated in Fig. 1. We consider the dataset *X* as an input, sampled from an underlying space ${\mathcal {M}}$ with holes and a non-linear and non-convex structure. We will rely on manifold learning^18^ to capture the topology, i.e., the shape, of this dataset’s underlying space. Manifold learning techniques assume that the points from the dataset *X* lie on an underlying manifold, i.e., the space ${\mathcal {M}}$^19^. A manifold, which is intuitively a generalization of the notion of a curved surface, is a topological space that locally behaves as a Euclidean space but may have different global properties. Many algorithms approximate the underlying manifold structure of a dataset *X* by constructing the nearest neighbor graph^11,18,20^. Among others, the algorithms PCA^21^ and MDS^22^ aim to approximate a supposed linear manifold from a dataset *X*, while the algorithms Isomap^23^, Local Linear Embedding^24^, Laplacian Eigenmaps^25^, Hessian Eigenmaps, TSNE^26^ and UMAP^11^ aim to approximate non-linear manifolds. In this work, we assume ${\mathcal {M}}$ is a non-linear Riemannian manifold. Inspired by existing techniques from the nearest neighbor construction step of Isomap^23^ and UMAP^11^, we propose to build a nearest neighbor graph *G* to approximate the manifold ${\mathcal {M}}$ and its topology, whose vertices are the points of *X* and where an edge exists between two points if one of them is among the *k* nearest neighbors of the other in the ambient space ${\mathcal {A}}$. In order to enable the computation of distances and geodesics between all points, we add *connecting edges* to the graph when it is composed of disconnected components. We store the status of those edges and discard them when considering the shape of the datascape to avoid disrupting the topological properties of the underlying manifold. The graph is made undirected, thereby establishing a reciprocal notion of neighborhood between points (see “Materials and methods”, section ‘Approximating the underlying manifold M with a neighborhood graph G’ for details on the connecting component and symmetrization steps). We equip the edges of *G* with weights corresponding to the distance between two nodes measured using the metric of the ambient space ${\mathcal {A}}$ (see Fig. 1b,c, and “Materials and methods” for details). The metric of the ambient space is crucial since it determines how the closest neighbors are chosen. Typically, manifold learning algorithms opt for Euclidean distance; however, users can select a distance metric that aligns more closely with the similarity among their data points, considering the study’s context and the nature of the data being processed.

The graph *G* captures the topology of the underlying space ${\mathcal {M}}$ and associates to each point of the dataset *X* a set of nearest-neighbor points called its neighborhood. However, *G* alone does not capture (a) the proper shape of the underlying space ${\mathcal {M}}$ and (b) the natural uncertainties of the data as it does not provide a notion of volume, interior or exterior.

A naive approximation of a dataset’s shape consists of building the dataset’s convex hull. However, unless the space ${\mathcal {M}}$ is convex, approximating ${\mathcal {M}}$ with a convex hull makes holes and cavities vanish, resulting in a loss of topological structures and information. Instead, we advocate for generalizing the above approach using the concept of Algebraic Topology called a *good cover*^27^. A cover of a space is a family of sub-spaces whose union is the space itself. This family is a good cover if all the intersections of the sub-spaces are convex. Having convex sub-spaces is a sufficient condition. Intuitively, a complex shape, can be decomposed into smaller and simpler convex shapes. We want to equip each points of *X* with a subspace of ${\mathcal {A}}$ that will account for its neighborhood and describes a sort of sampling uncertainty around it. The union of these subspaces forms the shape of the datascape. In Fig. 1c, one can see that the dataset *X* is included inside an oversized shape, delimited by the most exterior blue edges. This shape is the union of small polyhedrons formed by the graph’s edges *G*. These convex polyhedrons are the convex hulls of the neighborhoods of the vertices *X* of the graph *G*. Therefore, we approximate the neighborhood of every point of *X* by the convex hull of its graph neighbors, denoting it as *local convex hull* or *neighborhood convex hull*. The overall shape and volume of ${\mathcal {M}}$ is then approximated by the union of all these hulls, respecting the manifold topology of the graph *G*. In addition, to approximate the boundary surface of the dataspace ${\mathcal {M}}$, we identify the points that lie on this surface—the extreme points ${\mathcal {E}}$—as the points part of the union of all local convex hulls which are extreme in every local convex hull they lie in. We are especially interested by the points of *X* lying on this surface. For illustration in Fig. 1c, the point *C* is not extreme in its convex hull, whereas *D* is extreme in its convex hull and extreme or outside every other convex hull. This property yields another advantage: it allows assessing if a new point is inside (resp. outside) the datascape by checking if it is located inside any (resp. outside every) local convex hull (Fig. 1d). A new point sampled on ${\mathcal {M}}$ but not located inside the datascape might reveal interesting, not yet captured, information about ${\mathcal {M}}$ . We also can measure how extreme a point is by measuring its distance to an extreme point ${\mathcal {E}}$. Finally, *G* allows one to estimate topologically valid paths between a pair of points of *X*, and related distances as the sum of the weights of the edges of the shortest path, called geodesic, between them (Fig.  1c, for the shortest path and distance between A and B). The datascape ${\mathcal {D}}$ is a combination of the k-nearest neighbor graph *G* that approximates the underlying dataspace manifold ${\mathcal {M}}$ on which lies the dataset *X*, a set of extreme points ${\mathcal {E}}$ which defines its border and a set of convex hulls whose union form the shape of the datascape.

### Datascape of a simulated dataset

**Figure 2:**
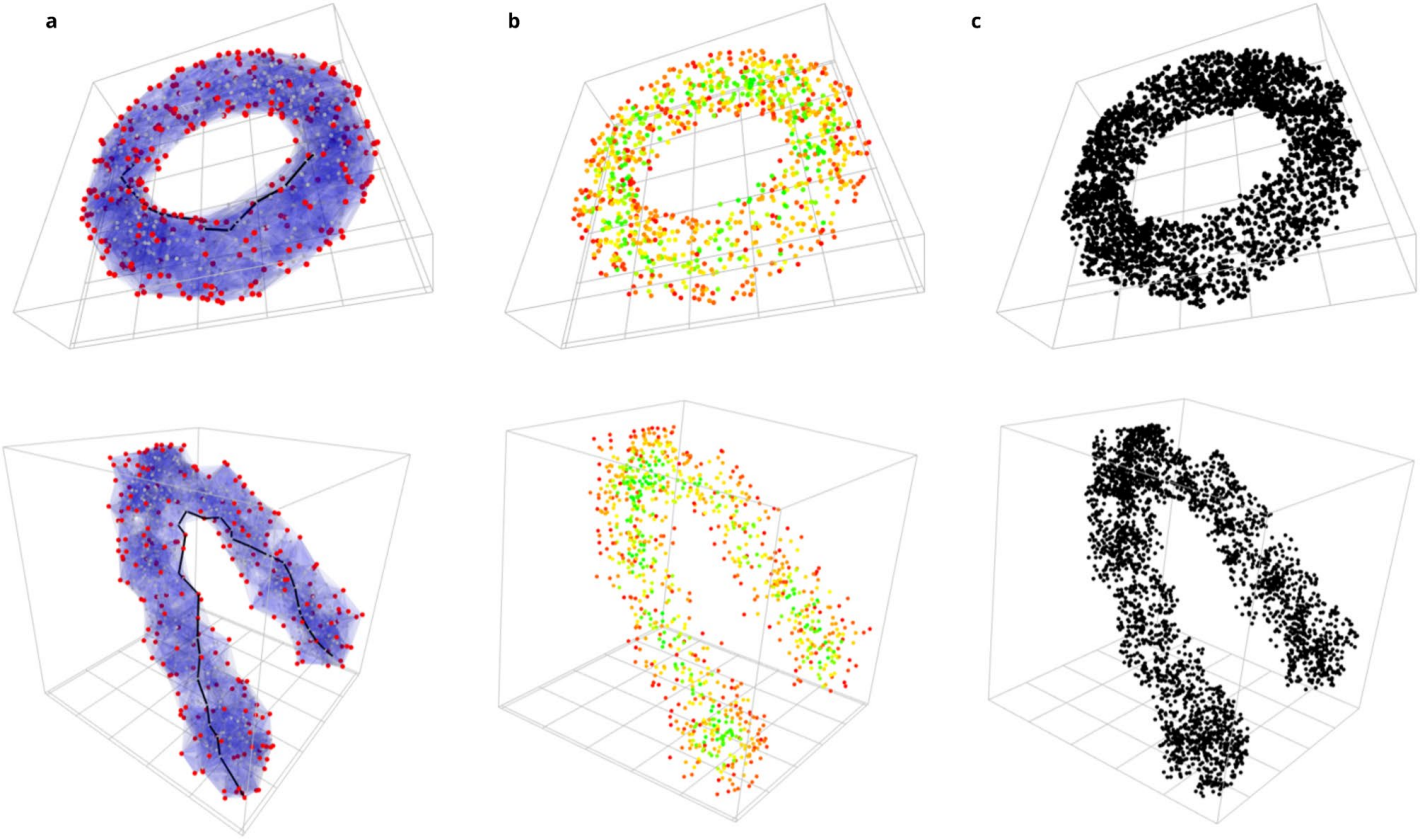
Illustration of the Datascape’s application on simulated datasets in 3d. (**a**) Datascape of simulated datasets. The neighborhood of each point, approximated by a convex hull, is depicted in light blue. The points identified as extreme, i.e., lying on the shape’s surface, are colored in red. The points inside the shape are grey. The black line depicts the shortest path (i.e., geodesic) between two faraway points in the datascape. (**b**) We colored each point for its proximity to an extreme point of the datascape. The points the farthest from extreme points are colored in green, whereas the red points are the closest. (**c**) Random sampling of 3000 points sampled inside the datascape showing that the shape is preserved.

For preliminary application, we generated 1000 samples from a mathematical formulation that incorporates noise around a given function (see “Materials and methods” for details and Fig. 2) and inside a torus. We constructed the corresponding datascapes with a k-nearest-neighbor graph (k = 10). Figure 2a depicts the neighborhood convex hulls, whose union approximates the data space, in blue and the datascape extreme points in red. It is worth noticing that extreme points are accurately located around the shape of the data. Figure 2a also depicts a geodesic between two points that follows the shape of the data. Measuring a distance along this geodesic gives a good approximation of the distance in the space of the dataset. Figure 2b underlines each point given their proximity to an extreme point of the datascape and emphasize a backbone that accurately approximates the function behind the sinusoidal data and highlights the topological structure of the torus. We artificially generated further points within the datascape with a triple density compared to the first estimation to further assess the precision (i.e., we randomly sampled three points in every neighborhood convex hull); see Fig. 2c for illustration, sustaining a similar shape as the original one. This latter property is potentially interesting for imputing missing heterogeneous data  (see reference Supplementary Materials for discussion) or generating synthetic data.

**Figure 3:**
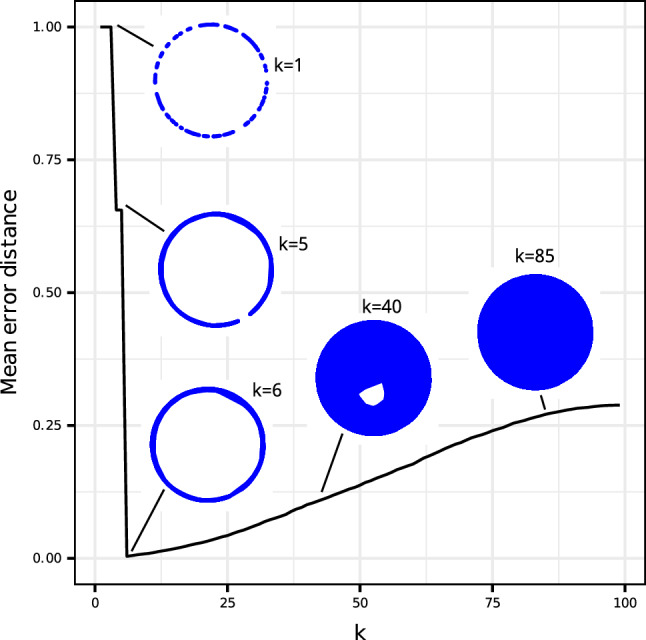
Illustration of the progression of the average error in distances measured within the datascape (without connecting the graph components) and the theoretical distance of the underlying manifold (in this case, a circle) for a given value of *k*. Specific values of *k* were selected, and the resulting shape and topology of the datascape were included in the visualization.

To investigate the influence of the parameter *k* on both the overall shape of the datascape and the accuracy of approximating the true metric of ${\mathcal {M}}$, we conducted a study by sampling 100 points on a unit circle and constructing various datascapes with *k* values ranging from 1 to 100. The comparison involved assessing the theoretical distances on the circle between every pair of points against the distances measured within the datascape. The evolution of these distances, depicted in Fig. 3, reveals a non-smooth pattern, exhibiting variations corresponding to key topological changes induced by different values of *k*. Very small values of *k* create several components (before connecting these components) in the datascape ($k=1$), where no distance can be calculated. For the sake of visualization, we standardized the error distance associated with the infinite distance in the graph with a constant value of 1, which remains maximal in this graph. For $k=4$, the datascape becomes a single component with no cycle until $k=5$, and the mean error distance drops significantly. However, we can see in the shape of the datascape with $k=5$, that points close to each other on the circle remain, due to sampling noise, abnormally distant in the datascape. The topology of the circle appears at $k=6$, which leads to the smallest error distance of the plot. The distance approximation in the datascape is the best when the datascape is equipped with the key topological features of the circle manifold. Therefore, finding an akin value of *k* is an important step in building the datascape. To establish a systematic method for selecting *k* in more general cases and higher dimensions, we propose a topological data analysis of the dataset *X* based on persistent homology and persistent diagrams. Details of this methodology are available in the Supplementary Materials section.

### Spatial dataset: clustering and geodesic analysis of the global ocean

Ocean studies tackle some of the most significant spatial data. This field relies on a profound understanding of the Earth’s oceans, which hinges upon our ability to dissect and interpret critical measurements encompassing temperature, salinity, and water mass momentum. In stark contrast to atmosphere studies, delving into the intricate mechanisms of the inner ocean presents a challenge, necessitating a heavy reliance on the analysis of in situ observations and measurements. As a significant achievement, unraveling the Lagrangian circulation principle, where space is intricately intertwined with time, becomes pivotal in comprehending the dynamic nature of oceanic phenomena (i.e., the seascape)^28^. Furthermore, analyzing these data leads to identifying distinct marine provinces, as exemplified by the Longhurst system, which aids in categorizing and comprehending the vastness of oceanic ecosystems^29^. Remark that in such a global ocean context, topological analysis has emerged as a valuable tool, facilitating the comparison of models and observations^30^. However, these global ocean scale advancements do not fully integrate recent strides in biological research, such as high-throughput sequencing. Addressing this gap, the Tara Oceans campaign emerges as a groundbreaking initiative, aiming to incorporate cutting-edge biological techniques into oceanographic exploration^31^. With a focus on exploring the extreme states of the global ocean, Tara Ocean holds the promise of better understanding the intricate interplay between physical and biological components within the epipelagic layer of the ocean^32^.

**Figure 4:**
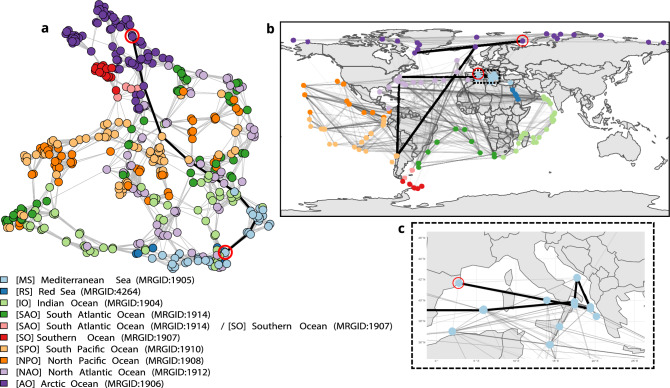
Tara Oceans metadata datascape. (**a**) Graph of the datascape computed on the Tara Oceans metadata dataset (i.e., projected in two dimensions for the sake of visualization using the Fruchterman–Reingold layout algorithm). Each node is a sample colored by ocean province, and edges connect neighbor samples in the datascape. The bold line shows the shortest path (i.e., geodesic) between a point in the western Mediterranean Sea and a point in the Arctic Ocean, both circled in red. (**b**) The datascape and the geodesic are projected on the planisphere, emphasizing k-nearest-neighbor edges from the datascape graph. Two stations linked on the map are neighbors in the datascape. Colors are proportional to the distance (i.e., the darker lines are smaller distances in the datascape). The map has been created with R version 4.1.2 (https://www.r-project.org/). (**c**) Zoom on the Mediterranean Sea.

We computed the datascape of the Tara Oceans dataset for a preliminary application in ocean studies and identifying connectivity between samples that describe extreme statuses of the ocean. The Tara Oceans dataset, consisting of approximately 900 ecological stations, measures nearly 90 environmental parameters across the global ocean^33^, collected over several years during two oceanographic campaigns. This application aims to classify ecological stations based on their biochemical properties and compare this clustering with a meta-genomic analysis^34^. We constructed the datascape over several variables (i.e., temperature, nitrate and iron concentrations, oxygen, salinity, chlorophyll A, Light, the flux of Carbon at 150 m depth—see^33^ for details and units). Our analysis confirms that ocean currents (i.e., Lagrangian connectivities) significantly impact station similarities, as observed via the sole genomic observations^35^. We thus identified that stations in the same ocean or province belong to the same neighborhoods (higher connections between samples from similar colors). The datascape can, therefore, capture known similarities between geographic regions. Still, the connections between stations in the datascape show that geographically, two extreme stations far apart can be similar (see Fig. 4). In particular, it shows that similar ocean states are linked, like water masses at similar latitudes separated by a continent (i.e., horizontal lines in Fig. 4b) or polar samples linked across the globe (i.e., Southern Ocean samples in red are connected to the Artic ocean samples in purple).

As the Tara Oceans datascape relies on extreme ocean state samples at a global scale (i.e., few mesoscale samples), investigating geodesics between these states is also insightful. For illustration, the datascape reveals that the shortest path between two stations does not necessarily follow natural geographic or Lagrangian trajectories, as illustrated by the bold geodesics connecting the western Mediterranean Sea to the Arctic Ocean in Fig. 4b,c. It proposes the smoothest similarity path between the two stations, which connect water masses from the Humboldt Current and those from the Gulf Stream, therefore showing a process of *Atlantification* of the Arctic Ocean that results from climate change^36^. Our findings provide new insights into the Tara dataset and highlight the potential of the datascape framework for analyzing and visualizing large, complex datasets, but call for further analysis, including incorporating longitudinal datasets, metagenomics knowledge, or Lagrangian circulation across the globe to enrich previous studies that define the concept of the seascape^34^.

### Longitudinal dataset: application to health monitoring

**Figure 5:**
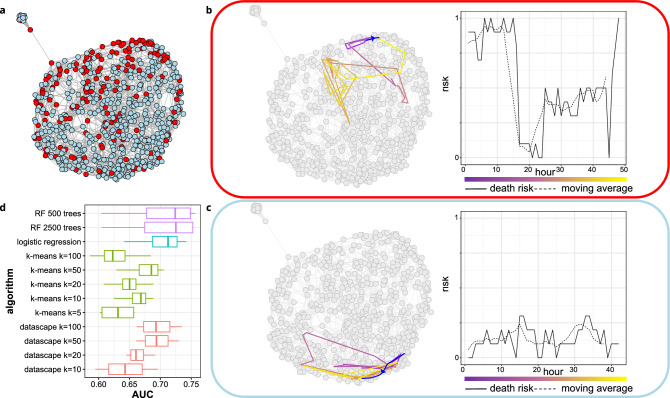
Physionet datascape. (**a**) Graph of the datascape on the clinical data measured at the 48th hour. The graph is projected in two dimensions thanks to the Fruchterman–Reingold layout algorithm. The red nodes represent deceased patients, whereas the blue ones are those in remission. (**b,c**) On the left, visualization of the trajectories of resp. a deceased patient and a patient in remission within the datascape over time. The color of the trajectory evolves from purple (1st hour of hospitalization) to yellow (48th hour of hospitalization). The blue lines represent the geodesic between the 1st and the 48th hour of hospitalization—the corresponding evolution of the ratio of deceased patients in the neighborhood of the studied patients pictured on the right panel. (**d**) Benchmark on 4-fold model training, comparing the distribution of the AUC of the different models benchmarked. The predictive model based on the datascape is trained with several values for the parameter k.

We computed the datascape of clinical data available for the Physionet cardiology challenge^37^. The data aims to monitor patients’ clinical parameters for prediction and personalized medicine over time. Figure 5a illustrates the resulting datascape, where nodes (patient data) are colored by category: deceased patients are in red, whereas patients in remission are in blue. As the Physionet datascape shows regions relatively specific to the patient’s status, we aim to experiment how the datascape performs as an unsupervised classifier. For this purpose, we associate a risk score for each patient (i.e., node) by calculating the ratio of deceased patients in its neighborhood (see “Materials and methods” for details). It shows that a patient in the upper region of the datascape has a neighborhood enriched with deceased patients compared to the lower region. We computed several datascapes with different values for the parameter *k* to assess this putative predictive feature further. We compared the distribution of AUC (area under curve) obtained over a 4-fold with the results of supervised predictive algorithms; two diverse random forests^38^ and a logistic regression (see Fig. 5d); and the unsupervised method k-means, which separates the space in *k* cluster, in a similar manner the datascape does with local convex hulls. In this context of building a classifier, *k* should be chosen so that it maximizes the given objective: the AUC. All algorithms have been trained on a train dataset and evaluated on a test dataset. As expected, both supervised algorithm show better AUCs than the unsupervised algorithm, datascape included. However, we can see that with *k* = 50, the sole datascape structure shows better AUCs than the 5 different *k*-means algorithms. Overall, it shows that an unsupervised construction of the datascape captures accurate differences between the two populations of patients. As its structure is relevant for unsupervised prediction, we monitored the trajectory of a patient within the datascape and its formalization via geodesics. For illustration, Fig. 5b,c illustrate the trajectories of resp a deceased patient and a patient in remission. The patients evolve in a datascape region enriched with patients from the same category. Furthermore, the patient’s trajectory does not follow a geodesic (blues arrows in Fig. 5b,c) between the vertex of the first hour of stay and the vertex of the last hour of stay. Our study illustrates how valuable the datascape framework is to studying multidimensional longitudinal data regarding an existing reference space and, with the same abstraction, its capacity to leverage graph theory to build predictive models.

## Discussion

The datascape abstracts a dataset using a neighbor graph and the union of convex hulls. The construction of the neighbor graph depends (1) on the choice of an ambient metric and (2) on the value of a parameter *k* used for initializing the number of neighbors for each vertex in the graph. The choice of the ambient metric is crucial in our work since this is how we determine the closest neighbors. Choosing a different metric might result in a different structure of the datascape and different weights on all edges. We have chosen the Euclidean distance as the ambient metric for applications presented herein. Still, the construction of the datascape is generic, and other metrics could be used given the context of the user’s study, such as the Manhattan distance, the Mahalanobis distances, or distances adapted to handle qualitative datasets^39,40^. However, one must carefully select a metric defined in the ambient space and that suits the nature of the different dimensions of the data. We illustrate this fact with the Wasserstein distance which requires a specific data type and an assumption about the sampled data *X*. The Wasserstein distance allows to measure the distance between two probability distributions over a manifold. The underlying concept involves measuring the work needed to transport the mass from a distribution A onto a distribution B to obtain distribution B. The transport should respect the geometry, shape, and metric of the probability space. Therefore, to use the Wasserstein distance instead of the Euclidean metric in the general case, (a) the data must contain a probability distribution as a dimension and (b) the underlying manifold has to be known, or an assumption about it has to be made—such as when the user is aware that the data is sampled over a sphere. However, in the general case, the underlying manifold remains unknown leading us to assume that the data is sampled over ℝ^n^, and we aim to approximate it through the datascape. In the case of data points *X* with dimensions representing known and probabilized manifolds, i.e. a probability distribution, the Wasserstein distance could be combined with the Euclidean distance or other metrics to build a meaningful distance given the heterogeneity of the dimensions. At last, The Wasserstein distance could be used on a probabilized datascape, once already constructed.

The choice of *k* is also context-dependent, as it affects the ability of the abstraction we build (the datascape) to preserve the geometric and topological properties of the underlying space ${\mathcal {M}}$. We studied in Fig. 3 how the choice of *k* affects the approximation of the metric of ${\mathcal {M}}$ and we proposed in Supplementary Materials a topological data analysis pipeline to choose an akin value of *k* to capture key topological features of ${\mathcal {M}}$. However, the underlying manifold ${\mathcal {M}}$ being unknown, we still cannot guarantee that the datascape will be equipped will all the key topological features of ${\mathcal {M}}$. Techniques have been developed to analyze the effect of *k* and to help point out the value of *k* that best preserves the underlying properties of ${\mathcal {M}}$. As an example, one can use topological data analysis such as persistent homology^41^ to study how the choice of *k* impacts the persistence of “shapes” in the abstraction we build of ${\mathcal {M}}$. It is easy to see that for *k* = 0, there will be as many components in the abstraction as there are points in the dataset we use, while for a large enough *k*, there will be only one component. The persistence of components and holes in the abstraction can be studied by a statistical analysis over the potential values of *k*: those with the longest duration of life (when *k* varies) are called stable topological properties. The ultimate choice of the parameter *k* should therefore be guided by how the obtained datascape captures those stable topological properties. Persistent diagrams provide a tool to explore the duration of life of topological properties, and a statistical analysis to detect the more stable properties in a persistent diagram and their associated *k*’s will help to reveal an adequate value for the parameter *k* for a given dataset. However, such topological data analysis are usually computationally expensive and most of the users of data representation techniques that use a similar parameter *k* (UMAP, tSNE, PHATE,…) do not perform them and instead choose a trade-off value for *k*. In this work, we have often chosen $k = 10$ as a tradeoff between computation time and precision of the datascape. Still, as shown in Fig. 5d, the ideal value for *k* depends on the study’s objective and must be chosen by the user.

As for other seminal studies in data science, the datascape is subject to the curse of dimensionality. In the context of high-dimensional datasets, the amount of data needed to obtain a sufficiently dense space for further analysis increases exponentially with the number of dimensions. For the corresponding datascape, having too many dimensions concerning the data density might result in having all the points *X* located on the border, i.e., considered extreme, drastically limiting the interest in assessing whether a point is inside or outside the datascape. This issue is the motivation for applying a preliminary dimension reduction. In particular, dimension reduction (DR) algorithms^41^ have been developed to search for a projection of the data in a space with a smaller dimension with a minimal deformation for a given metric. Such algorithms can be used on datasets without prior knowledge of meaningful metrics. Thus, the metric obtained by building the graph of the datascape can be provided to a DR algorithm to pre-process the data. Once done, the datascape can be computed using the same metric (projected on the new space) without the dimensionality issue. The datascape does not allow us to distinguish between sparse regions and outliers: both are treated as zones in the graph where the density of points is lower than others. In the future, we could eventually envision techniques to detect whether a new point (that we want to include in the datascape) is far from any point already included, but even in this case we would not be able to decide whether this new point belongs to a legitimate region that has not been sampled yet or whether it is an outlier.

Before considering adding a new point in a given datascape, one might be interested in deciding whether this addition brings further information to the datascape. Suppose this point is outside the boundaries of the existing datascape. In that case, it will provide a new neighborhood and cover a so-far unexplored part of the space, making its addition meaningful in knowledge. Reversely, if this point is already inside the boundaries, its addition will only be significant if it seriously disrupts the geodesic structure of the datascape. To decide whether this is the case, one could compute the matrix of the pairwise geodesic distances of the points “around” this new point—which approximates the local Riemannian metric distance—before and after adding the new point in the datascape. The difference between those matrices will measure how disruptive the new point is in the datascape and whether its addition is meaningful. Remark that choosing a threshold for this measure is akin to finding the tradeoff between exploration and exploitation in learning techniques.

By abstracting the data, the datascape is a formal model of the dataset. In other words, it is a proxy that one can use to study the dataset itself, from its topology, uncertainties, and use for prediction. The datascape framework answers many problems already handled by existing methods. For example, a classical data science pipeline proposes generic steps embedded by the datascape^42–44^. These steps start by imputing data via imputation algorithms (i.e., MICE^45^ or ImputePCA^46^), exploring data via dimension reduction technique (i.e., PCA^47^, t-SNE^48^ or UMAP^11^), building a classification tool (i.e., HDBSCAN^49^ or SVM^50^) for identifying differences between samples by measuring their similarities through a metric^40^, building a predictive tool (i.e., neural-networks, random-forests, GLM) that is finally evaluated thanks to different indicators (i.e. MAE, RMSE, area under ROC curve^51,52^). However, while often used sequentially, each of the above steps is performed using different abstractions, whose compatibility is often not guaranteed or even tested. The datascape framework is as efficient as the above steps and allows a single abstraction to perform all, from data imputation, and dimensions reduction, to classification, prediction, data generation as well as topological data analysis^53^. As a benefit of an integrated framework, it provides the tool to measure the quality of the different operations thanks to a metric respecting the topology of the data.

Beyond the incremental construction of the datascape presented above and its direct use in data science, the datascape opens several perspectives that are now within reach after its formal definition. In particular, we are interested in other types of operations that could be done at the datascape level: the comparison, union, intersection, and addition of datascapes. Such operations are the ingredients of a so-called model- or component- algebra^54,55^, studied in different contexts. We advocate that using datascape could open the door to future work to better combine data with other modelings used in Life sciences^56^.

## Materials and methods

### Capturing the shape of a set of points *X*

Let $X=\{x_i\}_{i \in {\mathbb {N}}}$ a finite set of points in dimension *n*, sampled heterogeneously on ${\mathcal {M}}$, a compact Riemannian sub-manifold with a Riemannian metric *g* embedded in an ambient space ${\mathcal {A}}$ with a metric $d_{\mathcal {A}}$. In the following, we propose a graph *G* that approximates the underlying manifold ${\mathcal {M}}$ and allows us to measure reliable distances on ${\mathcal {M}}$. In the following section, we describe the construction of *G* inspired by the first step of the Isomap algorithm^23^. For theoretical justification on how a graph approximates a manifold see^11,57^.

#### Approximating the underlying manifold ${\mathcal {M}}$ with a neighborhood graph *G*

Let $G=(X,E)_{\omega }$ be an undirected weighted graph, where *X* are vertices, $E \subseteq X \times X$ are edges and $\omega : E \rightarrow {\mathbb {R}}^{+}$ is a weight function, assigning positive real-valued weights to the edges. In the following, we write $E_{ij}$ for the edge between $x_i$ and $x_j$, and $\omega _{ij}$ for its weight. We now briefly recall how the edges of the graph *G* are constructed in the first step of the algorithm from Isomap^23^. Let $k \in {\mathbb {N}}$ be a parameter that we will use for initializing the number of neighbors each vertex is connected to. Given *k*, for each $x_i$, we start by computing the *k* nearest neighbors of $x_i$ in *X* using the existing metric $d_{{\mathcal {A}}}$ of the ambient space. This step has a complexity of ${\mathcal {O}}((k+1) *n^2)$. This set is called the *k*-nearest natural neighbors of $x_i$ and is written ${\mathcal {N}}_{k}(x_i)$ = $\{x_{i_1},\ldots ,x_{i_k}\}$.

As we assume the points from *X* are heterogeneously sampled on the manifold ${\mathcal {M}}$, an isolated point $x_i$ can have as one of its *k*-nearest neighbors a point $x_j \in {\mathcal {N}}_{k}(x_i)$ that is located in a more dense region of *X*. As a consequence, it might be the case that $x_i \notin {\mathcal {N}}_{k}(x_j)$, meaning that the notion of *k*-nearest natural neighbor is not symmetrical. To establish symmetry in the notion of neighborhood, we enlarge the set of neighbors by adding to the natural neighbors what we will call the enforced neighbors ${\mathcal {N}}^{+}(x_i) = \{x_j \in X \ | \ \forall x_j\not \in {\mathcal {N}}_{k}(x_i), \ x_i \in {\mathcal {N}}_{k}(x_j)\}$.

This allows us to obtain a graph $G_0 = (X,E')$, where the symmetric edges $E'$ connect all vertices that are neighbors, i.e. $E_{ij} \in E' \iff x_j \in {\mathcal {N}}_{k}(x_i) \cup {\mathcal {N}}^{+}(x_i) \iff x_i \in {\mathcal {N}}_{k}(x_j) \cup {\mathcal {N}}^{+}(x_j)$.

Unfortunately, the graph $G_0$ is not necessarily connected; therefore, paths between every pair of points of *X* cannot be found. As a consequence, we use the following steps to connect the components to build a connected graph *G* from the graph $G_0$.

1. We start by identifying every maximal connected components in $G_0$ and call them $C_1, \ldots C_m$. This can be done in linear time w.r.t. the number of edges in the graph using a simple depth-first search.
2. Then, for every pair of connected components $(C_i, C_j)$, we identify a pair of points $\text{Con}_{i,j} = (x_i,x_j) \in C_i \times C_j$ that minimizes the pairwise distance using the ambient metric $d_{\mathcal {A}}$, i.e. such that
  $$\begin{aligned} d_{{\mathcal {A}}}(x_i,x_j) = \min _{x \in C_i, y\in C_j}d_{{\mathcal {A}}}(x,y) \end{aligned}$$
3. This allows us to define a set of connecting edges $E_{con} = \cup _{i,j \in \{1, \ldots m\}} \text{Con}_{i,j}$.
  However, this set is too large as it connects all connected components pairwise. We therefore consider the set $E_{suf}$ of all the subsets $E'_{con} \subseteq E_{con}$ that are sufficient for connecting the graph $G_0$, i.e., such that the graph $(X, E'\cup E'_{con})$ is connected, and choose among them the one that minimizes the total weight addition. This set is written $\text{Con}(G_0)$ and formally defined as follows:
  $$\begin{aligned} \text{Con}(G_0) = \mathop {\mathrm{arg\,min}}\limits _{E'_{con} \in E_{suf}} \left( \sum _{ t \in E'_{con}} d_{{\mathcal {A}}}(t) \right) \end{aligned}$$
4. Finally, we define the neighbors of a point $x_i \in X$ as
  1 $$\begin{aligned} {\mathcal {N}}(x_i) = {\mathcal {N}}_{k}(x_i) \ \cup \ {\mathcal {N}}^{+}(x_i). \end{aligned}$$
  and its connecting neighbors as
  2 $$\begin{aligned} {\mathcal {N}}_{\text{con}}(x_i) = \{x_j \ | \ (x_i,x_j) \in \text{Con}(G_0) \} \end{aligned}$$

We estimated that connecting *c* components of a graph with *n* vertices has a complexity of ${\mathcal {O}}(n^2 * (1+t) + c^3)$ with *t* the time to compute a pairwise distance with the ambient metric.

Using this construction, we obtain a connected graph $G = (X,E)$, where

$$\begin{aligned} E_{i,j} \in E \iff x_j \in {\mathcal {N}}(x_i) \ { \cup \ {\mathcal {N}}_{\text{con}}(x_i)} \iff x_i \in {\mathcal {N}}(x_j) \ {\cup \ {\mathcal {N}}_{\text{con}}(x_j)} \end{aligned}$$

Finally, we define the weight function of *G* as the distance given by the ambient metric $d_{{\mathcal {A}}}$ to points that are considered neighbors:

3 $$\begin{aligned} \omega _{i,j}:= \left\{ \begin{array}{ll} d_{\mathcal {A}}(x_i,x_j) &{} \text{ if } x_j \in {\mathcal {N}}(x_i) \ \cup \ {\mathcal {N}}_{\text{con}}(x_i) \\ \infty &{} \text{ otherwise. } \end{array} \right. \end{aligned}$$

The following explains how distances can be computed in the resulting weighted graph $G = (X,E)_\omega$.

#### Approximating distances on the manifold ${\mathcal {M}}$ with the length of the shortest path in the graph *G*

By construction, the weights of the edges in *G* represent the local distance between neighbors in the ambient space ${\mathcal {A}}$. Indeed, we assume that, locally, ${\mathcal {M}}$ has the same structure as ${\mathcal {A}}$ and the metric $d_{{\mathcal {A}}}$ is a local approximation of the metric of ${\mathcal {M}}$. However, the manifold ${\mathcal {M}}$’s shape might differ from the shape of ${\mathcal {A}}$. As a consequence, distances between non-neighboring points in ${\mathcal {M}}$ should not be computed using the ambient metric $d_{{\mathcal {A}}}$. On a manifold like ${\mathcal {M}}$, global distances should be computed along geodesics (i.e., along a straight line that follows the shape of ${\mathcal {M}}$). Since the purpose of the graph *G* is to abstract the structure of ${\mathcal {M}}$, geodesics of ${\mathcal {M}}$ should correspond to shortest paths in *G*. We, therefore, define a global notion of distance in *G* based on the shortest cumulative local (i.e. ambient) distance between neighbors.

Formally, let $x_i, x_j \in X$. A path $\rho$ between $x_i$ and $x_j$ is a sequence $\rho = e_1, \ldots e_m$ of edges, where $e_k = (x^k_l, x^k_r)$ and such that $x^1_l = x_i$, $x^m_r = x_j$ and for all $1 \le k \le m-1$, $x^k_r = x^{k+1}_l$. The cumulative weight of the path $\rho$ is the sum of the weights of its edges: $\omega (\rho ) = \sum _{k=1}^m \omega (e_k)$. Let $\Gamma _{i,j}$ be the set of all paths in *G* from $x_i$ to $x_j$. The geodesic distance $d_G$ between $x_i$ and $x_j$ in *G* is then defined as

4 $$\begin{aligned} d_G(x_i,x_j) = \min _{\rho \in \Gamma _{i,j}} \omega (\rho ) \end{aligned}$$

In practice, the shortest path can be found using state-of-the-art graph algorithms, such as Dijkstra^58^.

The length of such a path *p* is $\sum _{i=1}^{n_{p}-1} \omega (e_i)$.

### Identifying the shape and the boundary of the datascape

Now that we approximated the topology of the manifold ${\mathcal {M}}$ by a graph *G* and defined a metric that can be used to measure distances between points on this manifold, we propose a way to approximate the space spanned by the dataset *X* and its border. We call this space the shape of the datascape $\text{shape}({\mathcal {D}})$. The shape of the datascape should respect topological features of the manifold ${\mathcal {M}}$ such as holes and cavities.

**Figure 6:**
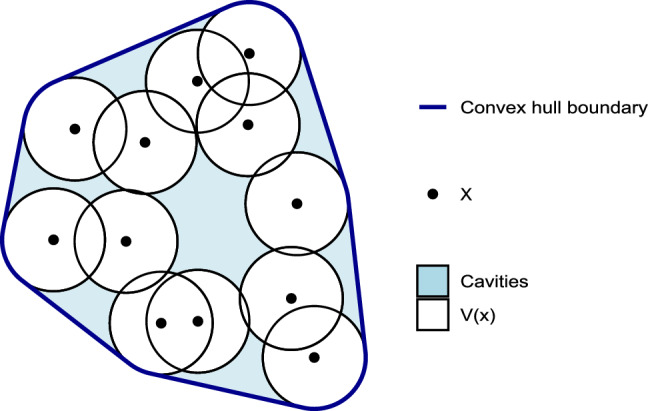
Set of points *X*, its convex sets *V*(*x*) and the cavities associated.

The notion of cavity of a dataset *X* is inspired by the convex deficiency of a set of points^59^. Let $x\in X$ a data-point. Let *V*(*x*) be a convex subset of the ambient space ${\mathcal {A}}$ such that $x \in V(x)$. Let $\text{Hull}(X)$ be the convex hull of $\bigcup _{x\in X}{V(x)}$. We define as cavities $\text{Cav}(X)$ the $y \in {\mathcal {A}}$ such that $y \in \text{Hull}(X)$ and $y \not \in \bigcup _{x\in X}{V(x)}$. We propose a visualization of this definition in Fig. 6.

From this definition, we see that defining the convex sets *V*(*x*) is key to constructing the shape of the datascape that takes into account topological features of the underlying space ${\mathcal {M}}$. Furthermore, as the graph defined in the previous section already captures, given the parameter *k*, the topology of the space ${\mathcal {M}}$, we seek to define the convex sets *V*(*x*) based on that graph.

A cover of a space is a family of sub-spaces whose union is the space itself. This family is a good cover if all the sub-spaces intersect are convex; having convex sub-spaces is a sufficient condition. Intuitively, a concave space can be seen as the union of smaller convex spaces. In the following, we, therefore, use convex hulls built on the neighborhoods of the graph’s vertices *G* as the elementary convex spaces to approximate the concave space containing the points *X*. Given a vertex $x \in X$, we define *V*(*x*) as the convex hull $\text{conv}({\mathcal {N}}(x))$ built upon the natural and enforced neighbors of *x* and call it the local convex hull of *x*. The connecting neighbors are not used to build the hulls so that the shape of the datascape remains composed of disconnected components (when this is the case) and respects the topology of the data. We define the shape of the datascape ${\mathcal {D}}$ as the union of all the local convex hulls. Computing the local convex hull algorithm thanks to the quickhull algorithm^60^ for every points of *X* has an expected time complexity of ${\mathcal {O}}(n * k^{d/2} / [d/2] !)$ with *d* the dimension of the data and *k* the chosen number of neighbors.

Formally:

5 $$\begin{aligned} \text{shape}({\mathcal {D}}) = \bigcup _{x\in X} \text{conv}({\mathcal {N}}(x)) \end{aligned}$$

We define the boundary of the datascape as the points located on the surface of $\text{shape}({\mathcal {D}})$. The points located on the surface of a convex hull $\text{conv}(x)$ are called extreme points and noted ${\mathcal {E}}(\text{conv}(x))$ and are defined as the smallest set of points of ${\mathcal {N}}(x)$ such that $\text{conv}({\mathcal {E}}({\mathcal {N}}(x))) = \text{conv}({\mathcal {N}}(x))$. As the different convex hulls might intersect, the extreme points of the hull of a point *y*, written ${\mathcal {E}}_y$, might be inside the convex hull of another point *z* and thereby not globally extreme. To make sure a point is globally extreme, we define the surface of the datascape, i.e., the extreme points of the datascape, as the following set of points:

6 $$\begin{aligned} {\mathcal {E}}({\mathcal {D}}) = \{ t \in \text{shape}({\mathcal {D}}) \ | \ \forall x \in X, t \in \text{conv}({\mathcal {N}}(x)) \iff t \in {\mathcal {E}}_x\ \} \end{aligned}$$

To reduce computation time in practice, we approximate the set of extreme points ${\mathcal {E}}({\mathcal {D}})$ by the set ${\mathcal {E}}'({\mathcal {D}})$ that reduces the search of extreme points among the points of *X*. For all $x \in X$, to check if *x* is globally extreme, we check if *x* is extreme in all the convex hulls of the datascape, or a subset of convex hulls containing *x*. Formally,

7 $$\begin{aligned} {\mathcal {E}}({\mathcal {D}}) = \{ t \in \text{shape}({\mathcal {D}}) \cap X \ | \ \forall x \in X, t \in \text{conv}({\mathcal {N}}(x)) \iff t \in {\mathcal {E}}_x\ \} \end{aligned}$$

In Fig. 1c, we draw the local convex hull corresponding to the neighborhood of the point *D*, which is extreme in this local convex hull and extreme globally at the datascape scale. On the contrary, the point *C* at the right of the datascape, whose neighborhood is represented by the red convex hull, is not an extreme point of the latter. Therefore this point is not a global extreme point. When implementing this methodology on simulated data in Fig. 1a, we successfully identify the points located at the 3D border of the sinusoidal shape and the torus.

Now that we have defined a methodology to approximate the shape of the datascape $\text{shape}({\mathcal {D}})$ and to identify the extreme points that lie on the border of this shape, we show how to use this to characterize a new data point in regard of the datascape.

### Characterizing a given point in the datascape ${\mathcal {D}}$

#### Measuring the distance between a given point and the bounds of ${\mathcal {D}}$

Once we have identified the set ${\mathcal {E}}$ of extreme points of the datascape, we are able, thanks to the metric $d_G$ defined earlier in (2), to compute the distance between a given point $x^*$ and the closest extreme point. The idea is to add $x^*$ in the datascape ${\mathcal {D}}$ by adding it to the graph *G*. Let $G^*$ be the graph based on *G* with the supplementary vertex $x^*$.

We will identify the *k*-nearest neighbors of $x^*$ and add the set of edges $E_{x^*}$ between $x^*$ and its neighbors $x^*_i, i\in {1,\ldots ,k}$ with weight $d_{\mathcal {A}}(x^*,x_i)$. Formally,

8 $$\begin{aligned} G_{x^*}=(X\cup x*,E \cup E_{x^*})_{\omega } \end{aligned}$$

In order to assess if $x^*$ is close to a border of ${\mathcal {D}}$, we compute the distance between $x^*$ and the closest extreme point:

9 $$\begin{aligned} d_{G}(x^*,{\mathcal {E}}):= \min _{\forall e \in {\mathcal {E}}} d_G(x^*,e) \end{aligned}$$

#### Assessing if a given point is inside or outside the datascape ${\mathcal {D}}$

The datascape is an approximation of the unknown manifold ${\mathcal {M}}$ based on the sample *X*. *X* being finite, it might provide an incomplete view of ${\mathcal {M}}$. Therefore, a new sample of ${\mathcal {M}}$, denoted as $X^*$, might help refine the datascape. A new point $x^* \in X^*$ is either inside the datascape, thereby bringing no further information to the datascape, or outside the datascape. The shape of the datascape $\text{shape}({\mathcal {D}})$ being a union of the neighborhoods of the set of points *X*, see Eq. (5), assessing if a point is inside or outside of the datascape is equivalent to determining if $x^*$ is inside any neighborhood or outside every neighborhood. This operation has a maximal time complexity of ${\mathcal {O}}(n^4)$. The datascape being a union of convex hulls, it is guaranteed to have an interior of dimension equal to or smaller than $k-1$. If *k* is equal to or smaller than the dimension of the ambient space ${\mathcal {A}}$ (or if the points in the dataset used for building the datascape are themselves on a hyper surface), the shape of the datascape is considered as an hyper-surface and being in the datascape is equivalent to being on that hyper-surface. Formally:

10 $$\begin{aligned}{} & {} x^* \in \text{shape}({\mathcal {D}}) \iff \exists \ x_j\in X, x^* \in \text{conv}({\mathcal {N}}(x_j)) \end{aligned}$$

11 $$\begin{aligned}{} & {} x^* \not \in \text{shape}({\mathcal {D}}) \iff \forall \ x_j \in X, x^* \not \in \text{conv}({\mathcal {N}}(x_j)) \end{aligned}$$

### Complexity analysis of building the datascape

In this section, we estimate the complexity of the different steps of the construction of the datascape.

As a preliminary step, we first need to compute the pairwise distance matrix between every point of the dataset. With $n$ points, we have to compute $\mathcal{O}(n^2)$ distances. The complexity of computing a distance between two points depends on the metric used. For the Euclidean distance in dimension $d$, which we use here, the complexity is in $\mathcal{O}(d)$.

Once the distance matrix is obtained, identifying the $k$ nearest neighbors of a given point can be done in $\mathcal{O}(k \times n)$. Therefore computing the $k$-nearest neighbors of all points can be done in $\mathcal{O}(k\times n^2)$.

Symmetrizing the notion of neighbors involves checking, for each point $x$, if it belongs to its $k$ neighbors’ neighborhood, each one composed of $k$ points. Checking if an element belongs to an array of size $k$ has a time complexity of $\mathcal{O}(k)$. For a given point $x$ with $k$ neighbors, the symmetrization step has a time complexity of $\mathcal{O}(k^2)$. For the whole dataset, the symmetrization step therefore has a complexity of $\mathcal{O}(n \times k^2)$.

After obtaining the graph, identifying connected components can be done in $\mathcal{O}(n^2)$, for example with a depth-first search algorithm, resulting in at worst $n$ components.

Assuming there are $c$ components, (with $c \geq 2$), with roughly $\frac{n}{c}$ points each (worst case scenario), we need to compute the minimal distances between each pair of components. If we have access to the pairwise distance matrix computed at the first step of the algorithm, which has a space complexity of $\mathcal{O}(n^2)$, the time complexity to retrieve all the distances between two components is $\mathcal{O}((\frac{n}{c})^2 \times t_{access})$ with $t_{access}$ the time needed to access a specific cell in the pairwise distance matrix. If the pairwise distance matrix is not stored, the time complexity becomes $\mathcal{O}((\frac{n}{c})^2 \times t_{distance})$ with $t_{distance}$ the time needed to compute the distance between two points given the ambient metric chosen. Now that we have the pairwise distance of all points between the two components, we keep only the pair of points with minimum distance. Finding this pair of points has a complexity of $\mathcal{O}((\frac{n}{c})^2$. We repeat the previous tasks for the $\mathcal{O}(c^2)$ pair of components, which takes $\mathcal{O}(c^2 \times ((\frac{n}{c})^2  + (\frac{n}{c})^2 \times t_{distance})$ which writes $\mathcal{O}(n^2 * (1+t))$ with $t \in \{t_{access},t_{distance}\}$. In our naive implementation, we connect the $c$ component by picking the $c-1$ pair of points in a specific manner and by adding an associated connecting edge in the graph.

We start by connecting the two components with minimum pairwise distance which has a time complexity of $\mathcal{O}(c^2)$. Then, we update the records of component pairwise distances so that the two components that have just been connected have the same id. We need to traverse the array of pairwise distances and replace the id of one of the 2 components with the id of the other. This step has a time complexity of $\mathcal{O}(c^2)$. Then, we reiterate these two steps until all the components have the same id. A total of $c-1$ iteration is necessary to obtain a single remaining connected component. The overall complexity of this loop is thus in $\mathcal{O}(c^3)$. The total time complexity to connect $c$ components is therefore $\mathcal{O}(n^2 * (1+t) + c^3)$ with $t \in \{t_{access},t_{distance}\}$.

Regarding the quickhull algorithm, its expected time complexity for $k$ neighbors of a point $x$ is $\mathcal{O}(k \times \log(k))$ for $d \le 3$ and $\mathcal{O}(k^{d/2} / [d/2] !)$ otherwise^60^. For $n$ points in a dimension of at least 4, the complexity is $\mathcal{O}(n \times k^{d/2} / [d/2] !)$.

Finally, to assess if a point is inside or outside the datascape, we have to check if it belongs to any local convex hulls. In the worst case where a point is not in the datascape, we have to check the $n$ convex hulls. To check if a point is inside a convex hull, one has to check if the given point can be written as a linear combination of the $r$ vertices of the convex hull such that the coefficients are all positive and their sum equals one. To do so we solve a system of $d+1$ equations and $r$ unknown variables ($r$ being the number of extreme points in the convex hull) with a Gaussian elimination known to be of time complexity $\mathcal{O}(r^3)$. In our case, the hull is built on the $k$ nearest neighbors and the $e$ enforced neighbors of a given point, the complexity of checking if a point is inside a local convex hull is thus at most $\mathcal{O}((k+e)^3)$ but probably less because not all the $k+e$ points are extreme points of the hull. $k$ is constant but $e$ depends on the considered hull. Roughly approximating the worst case, we know that $k + e < n$, and therefore that the time complexity to check whether a point is in the datascape is at worst in $\mathcal{O}(n^4)$.

To summarize, the main complexities are:

1. compute the matrix distance: $\mathcal{O}(n^2)$
2. find the $k$-nearest neighbors: $\mathcal{O}(k \times n^2)$
3. connection of $c$ different components: $\mathcal{O}(n^2 \times (1+t) + c^3)$ with $t \in \{t_{access},t_{distance}\}$
4. all local hull computations with the quickhull algorithm: $\mathcal{O}(n \times k^{d/2} / [d/2] !)$
5. deciding if a point is inside the datascape or not: $\mathcal{O}(n^4)$

### Simulated data

The sinusoïdal shape is constructed from the generation of 1000 points in 3 dimensions of the form $(x + \epsilon _x,y + \epsilon _y,z+\epsilon _z)$ such as $x \sim {\mathbb {U}}(0,2*\pi ), y = cos(x), z = cos(x - 0.5)$ with $\epsilon _x \sim {\mathbb {U}}(-1,1)$, $\epsilon _y \sim {\mathbb {U}}(-0.3,0.3)$ and $\epsilon _z \sim {\mathbb {U}}(-0.3,0.3)$.

The torus shape is constructed from the generation of 1000 points in 3 dimensions thanks to the package $\textbf{alphashape3D}$ in R.

An R Notebook is available to reproduce an example with 200 points. The number of points simulated can be changed in the notebook available in the zenodo package.

### Tara Ocean contextual data set

We removed duplicate records of the dataset and focused on the following variables: temperature, nitrate and iron concentrations, oxygen, salinity, chlorophyll A, light, and Carbon flux at 150 m depth—see^33^ for details and units. We processed missing data thanks to an unweighted k-nearest neighbor imputation algorithm^61^ with $k=1$. Data are available in the /data folder of the associated repository. The script 02_script_tarascape_data_ingestion.R reads and cleans the data for further analysis. The script 03_script_tarascape_make_all_figures.R creates the different figures of the tarascape and the computation of the geodesics.

### The Physionet Computing in Cardiology Challenge 2012 Data set^37^

The Physionet Computing in Cardiology Challenge 2012 aims to develop methods for patient-specific prediction of in-hospital mortality. Up to 42 variables were recorded at least once during the first 48 hours after admission to the ICU. Six of these variables are general descriptors (collected on admission), and the remainder is time series, for which multiple observations may be available. We worked on the patients of ICUType = 3, i.e. patients admitted to the Medical ICU.

We focused our study on the clinical data that were the most measured. Missing longitudinal values were imputed thanks to a naive approach. For a given patient and a given variable, we considered that the missing values were equal to the last known value for this patient. For example, if a value for a clinical variable was only available at $t_0$ and $t_5$ for a given patient, we assumed that the values at $t_1$,$t_2$,$t_3$,$t_4$ were identical to the value at $t_0$.

To further reduce the dimension of the data, we selected only the most relevant parameters by calculating their odd ratio and only keeping those with a p-value lower than 0.01. The datascape was then built using the last available measure of each patient (i.e. at the 48th hour).

The physionet dataset needed for our study is available in the folder data/physionet.org/

The script 04_script_physionet_make_datascape_figures.R reads and cleans the dataset and constructs the datascape and the patient’s trajectory and geodesic. Full data of the physionet challenge are available here: https://physionet.org/content/challenge-2012/1.0.0/.

### Supplementary Information

Supplementary Information.

## Data Availability

The datasets used and/or analysed during the current study and the source code are available from the corresponding author on reasonable request at https://zenodo.org/record/8340362^62^.
