# Supplementary material for: Datascape: exploring heterogeneous dataspace
Source: Sci Rep. 2024 Apr 5;14:7041. doi: 10.1038/s41598-024-52493-7 (PMC10997776; doi:10.1038/s41598-024-52493-7)
Supplement: Supplementary file 1 — Supplementary Information. [file 41598_2024_52493_MOESM1_ESM.pdf]

Supplementary Materials

Handling missing data with the datascape

Handling missing data from a dataset is a common problem in machine learning. Several methods exist to impute missing values as reviewed in [1]. The herein datascape is based on a knn-graph, which makes it natural to pre-process missing data with a k-nearest neighbor imputation algorithm [2]. The k-nearest neighbor imputation algorithm imputes a missing dimension of a point x by averaging the values on that dimension of the k-th closest neighbors of x in a knn-graph built thanks to the available dimension of x. A formal definition of this algorithm is presented in the next paragraph.

The k-nearest neighbor imputation can overcome the missing data problem in a dataset and perform better than a randomForest imputation algorithm in some instances [1]. This supplementary section aims at showing the importance of choosing an imputation technique regarding the topology of the data. We advocate that an imputation technique should preserve the shape of a dataset. We study, in two dimensions, the case of a dataset sampled on a circle, which is not linear, and the case of a linear dataset built from a linear relationship between the two dimensions of the data.

Materials and method

We now formally describe the k-nearest neighbor imputation algorithm. Let $x = (x^1, x^2, ..., x^n)$ be a point in dimension n. Given an index $1 \leq i \leq n$, x^i is the i-th component of x. Let $\alpha \subset \{1, .., n\}$ be the strict subset of indices of the components of x which need to be imputed. Let $\mathcal{C}$ be a set of complete points. Thanks to the components of indices $\{1, ..., d\} \setminus \alpha$ of the set of points $x \cup \mathcal{C}$ we compute a knn-graph K. Let $\mathcal{N}_{x,k}$ be the k-th nearest-neighbors of x in the graph K. We write θ_i for the set consisting of the i-th components of all neighbor points in $\mathcal{N}_{x,k}$. Formally, we define:

$$\theta_i = \{z^i | z \in \mathcal{N}_{x,k}\}.$$

In order to impute the missing components of x, we use a generic function $f : \mathbb{R}^k \mapsto \mathbb{R}$. In practice, we use an unweighted mean function for f but any other function could be used instead. Once f is chosen, We impute the missing components of x in the following way:

$$\forall i \in \alpha, x^i = f(\theta_i)$$

To illustrate how missing data imputation impacts the data topology in 2 dimensions, we generated 10 000 points on a circle of radius 1 and 10 000 points of coordinates $(x, 2x + 3 + \epsilon)$ with $\epsilon \sim \mathcal{N}(0, 400)$ and $x = \{1, ..., 10000\}$. In order to simulate missing data, we then randomly chose two sets of 1 500 points (that might overlap) and erased the first coordinate of the points in the first set and the second coordinate of the points in the second set. Once this was done, we imputed the missing values with different techniques, from naive to state-of-the-art algorithms described below.

Imputation with a mean (resp. median) — imputation of the missing values by computing the mean (resp. the median) of the total set of available values on the considered dimension.

Imputation with uniform — imputation the missing values with a random value between the minimum and the maximum of the available values of the considered dimension.

Imputation with distribution — imputation of the missing values randomly from the distribution of the available values of the considered dimension.

knn-imputation — imputation of the missing values thanks to a knn-graph with $k \in \{1, 2, 5, 10\}$ and the function f described above.

MICE — imputation of the missing values thanks to a Multivariate Imputation by Chained Equations [3] with different numbers of multiple imputation $m \in \{5, 10\}$.

imputePCA — imputation of the missing values of a dataset with the Principal Components Analysis model [4].

Results

In Figure S1, we have considered the data set sampled on a circle and depicted the data that is imputed from the two sets where one coordinate has been erased (the points that have not been modified are not depicted). We see in this figure that the different imputation algorithms produce data with various shapes. Most algorithms fail to impute data with the same shape as the original data. The naive methods **Imputation with uniform** and **Imputation with distribution** create data of the shape of a square. The commonly used methods **Imputation with a mean (resp. median)** as well as the **imputePCA** algorithm produce data of the shape of a cross. However, the state-of-the-art algorithm **MICE**, with $m \in \{5, 10\}$ produces data with circular elements, as well as the knn-imputation algorithm with $k \in \{2, 5, 10\}$. The imputation algorithm that performs the

best, in terms of shape preservation, is the knn-imputation algorithm with $k = 1$. With this last algorithm, the reconstructed data follows the shape of the original data set.

In the linear case illustrated in Figure S2, the **MICE** algorithm performs well and produces data with a similar shape to the original data. This is also the case for the knn-algorithm with $k = 1$. The knn-algorithms with $k > 1$ produce imputed data with a narrower shape. The **imputePCA** algorithm uncovers the underlying noise-free linear functions but fails at imputing data with the shape of the original data. The commonly used methods **Imputation with a mean (resp. median)** produce data with the shape of a cross.

Conclusion

We illustrated that imputing missing data leads to new data points that do not especially fit the original data's topology and shape. The discrepancy between the shape of the imputed data and the shape of the original data is more significant in the case of a non-linear data set than in the linear case. In machine learning, the performance of imputation algorithms is measured thanks to the distance between the imputed points and the response points. However, this distance is mostly Euclidean, which does not fit the topology of non-linear generic data sets. Consequently, an algorithm could perform well regarding such distances and produce points that do not belong to the original data space, profoundly impacting the result over such pre-processed data. We suggest that future research into missing data should also evaluate the imputed data's topology to assess the imputation algorithms' efficiency.

Choosing a value of k to build a k-nn graph thanks to a topological data analysis of dataset

When constructing the datascape, a $k$nn-graph parameterized by a value k is created. This graph plays a key role in the final shape of the datascape and on the distance measured on it. The datascape aiming to approximate the underlying manifold $\mathcal{M}$ of the sample dataset X, the distances measured on it should approximate closely the distances measured on $\mathcal{M}$. However the true metric on $\mathcal{M}$ is in general unknown to us and a proxy is needed to infer this metric. The topology of $\mathcal{M}$ constrains how the distances are measured on $\mathcal{M}$ and we believe that capturing the topology of $\mathcal{M}$ in the manifold will allow us to approximate the true distances on $\mathcal{M}$ more precisely.

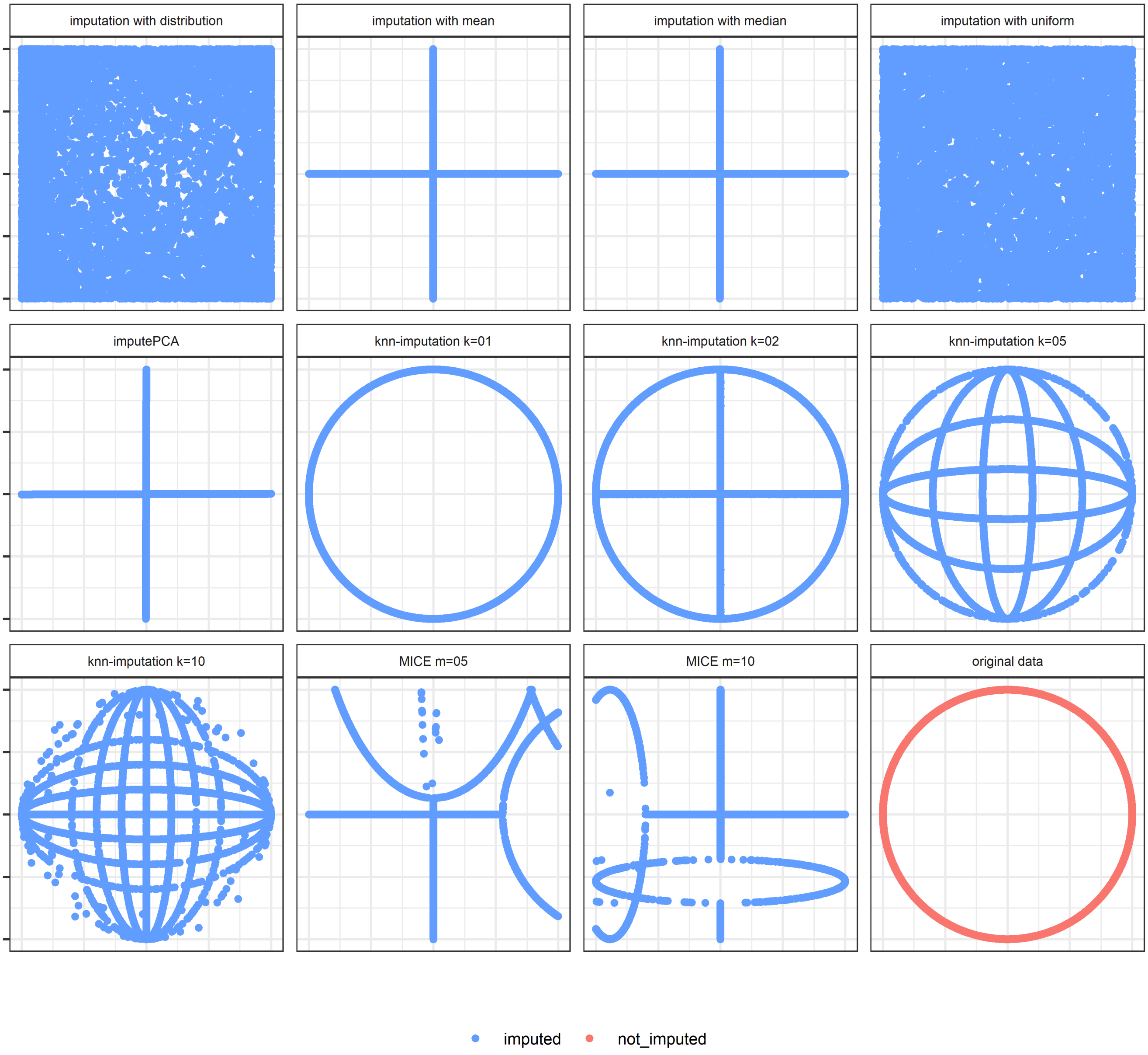

Figure S1: Imputation of missing data sampled on a circle. The bottom right box depicts the original data (before erasing one of their coordinates). In the other boxes, we depict the imputed data obtained with state of the art algorithms.

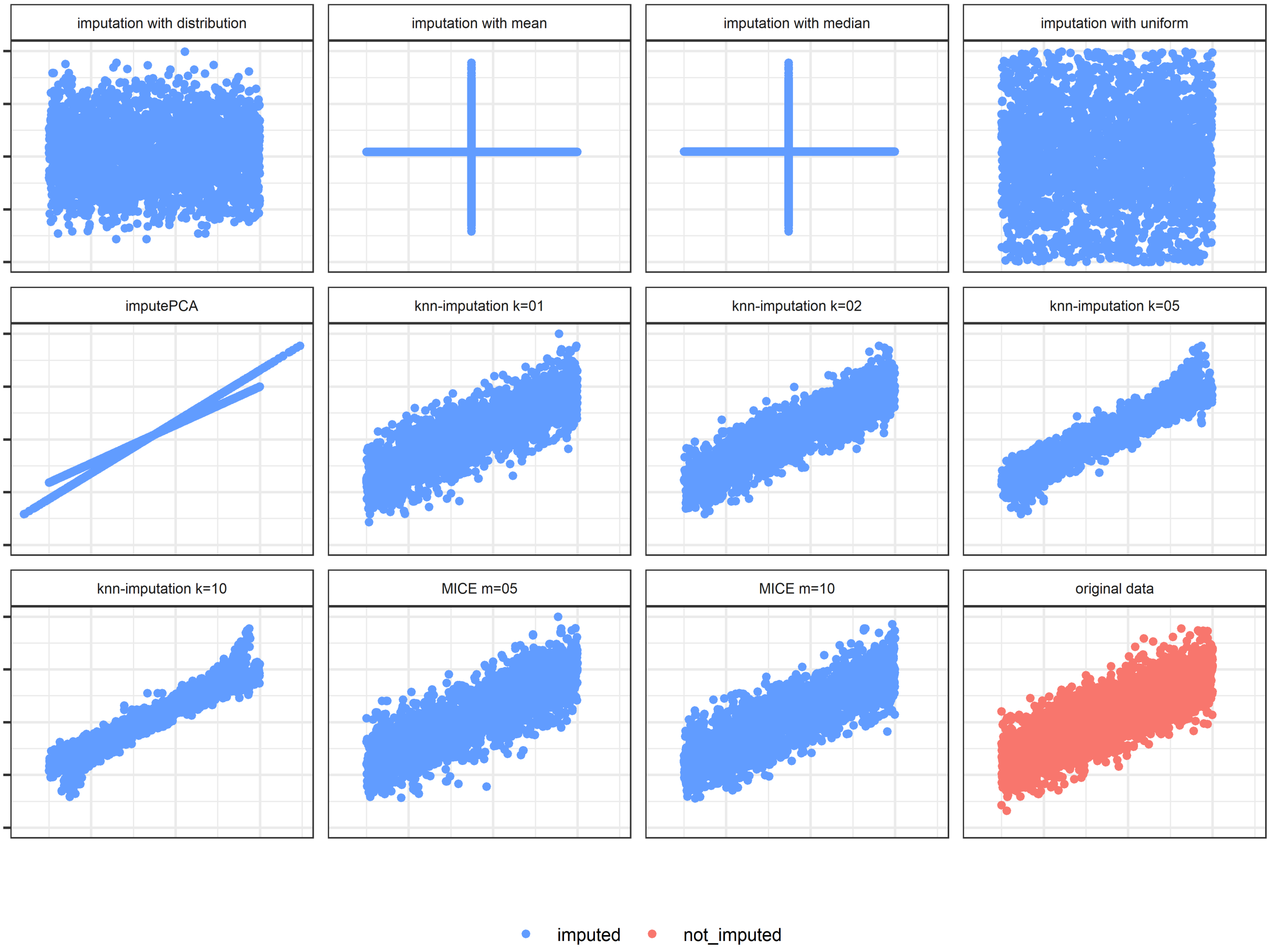

Figure S2: Imputation of missing data sampled on a linear dataset. The bottom right box depicts the original data (before erasing one of their coordinates). In the other boxes, we depict the imputed data obtained with state of the art algorithms.

However, when building k-nn graphs, the way one has to choose k is often eluded and no principled manner is described in algorithms such as ISOMAP, UMAP or PHATE [5, 6, 7] to choose an adequate or optimal value for k. In the following, we propose a method based on persistent homology and a persistent diagram, to choose a value for k. This value of k will allow us to build a $k$nn-neighbor graph G (before adding connecting edges) that captures the topology of $\mathcal{M}$ and therefore obtain an approximated metric close to the manifold metric.

Materials and methods

We generated a sample of 100 points on a circle depicted in Figure S3.a . We studied the persistent homology of this set of samples. To do so, we built a filtration on top of the dataset, which is a set of simplicial complexes parameterized by a value k, the k-nearest-neighbors in our case. For each value of k, topological features, as components and holes, are revealed through a persistent homology algorithm. For each topological feature identified, a record of its birth (resp. death), i.e. the value of filtration k at which it appears (resp. disappears), is recorded. This study has been performed thanks to the *rguhdi* package in the langage R. For more details on the persistent homology algorithm, persistent diagram and filtration, the reader can refer to [8, 9, 10].

Among the topological features denoted as $\mathcal{T}$, as revealed by a persistent diagram $\mathcal{PD}(X)$, a subset of $\mathcal{T}$ lacks specific topological significance and results from the inherent sampling noise in the data. Another subset, designated as $\mathcal{T}'$, encapsulates the fundamental topological structure and geometry of the data. In the case of a circle, for instance, $\mathcal{T}'$ would comprise the primary component and the hole. We propose a straightforward approach to identify $\mathcal{T}'$. To delineate two distinct clusters, we employ k-means on the set of persistence durations. The cluster with the highest mean duration is retained to constitute the subset $\mathcal{T}'$. Alternatively, other statistical methods could be employed to form a subset of stable $\mathcal{T}'$. Subsequently, we suggest selecting the minimum possible value of k that allows for the simultaneous existence of the most stable elements of $\mathcal{T}'$, determined by their persistence duration. This ensures that if there is no k value permitting the coexistence of all elements of $\mathcal{T}'$ in the graph, the

more stable ones are prioritized.

Results

The persistent diagram in Figure S3.b shows 4 groups of components with small persistence (birth at $k = 0$ and death between $k = 1$ and $k = 4$) which are considered as noise. The longest bar never disappears and represents the circle itself. We see at $k = 6$ the birth of a topological structure called a cycle (a topological hole) which dies at $k = 62$. The histogram of persistence duration in Figure S3.c shows two clusters, identified through a k-means algorithm, of topological features: many unstable ones with short persistence duration and two stable ones with persistence duration of 52 and 100. We depicted in Figure S3.d the datascape at the minimal value of k allowing coexistence of the two stable topological features. We observed in Figure ?? that this value of $k = 6$ minimizes the error between the distance measured on the datascape and on the circle. This study, which implied to build a simplicial complex for each k is computationally expensive, especially if the number of points is high. However, it shows us that a value of k between 6 and 62 allows us to build a datascape respecting the main topological features of the underlying manifold of the data.

Conclusion

We proposed a straightforward pipeline to choose an adequate value of k to construct a $k$nn-graph based on topological data analysis. In the studied example, the identified value of k equips the datascape with the most stable topological features and allows the best approximation of the unknown manifold metric. We believe further studies should be done to improve persistent homology in the context of the datascape, especially to choose, in high dimension, the most stable topological features among those highlighted by a persistent diagram.

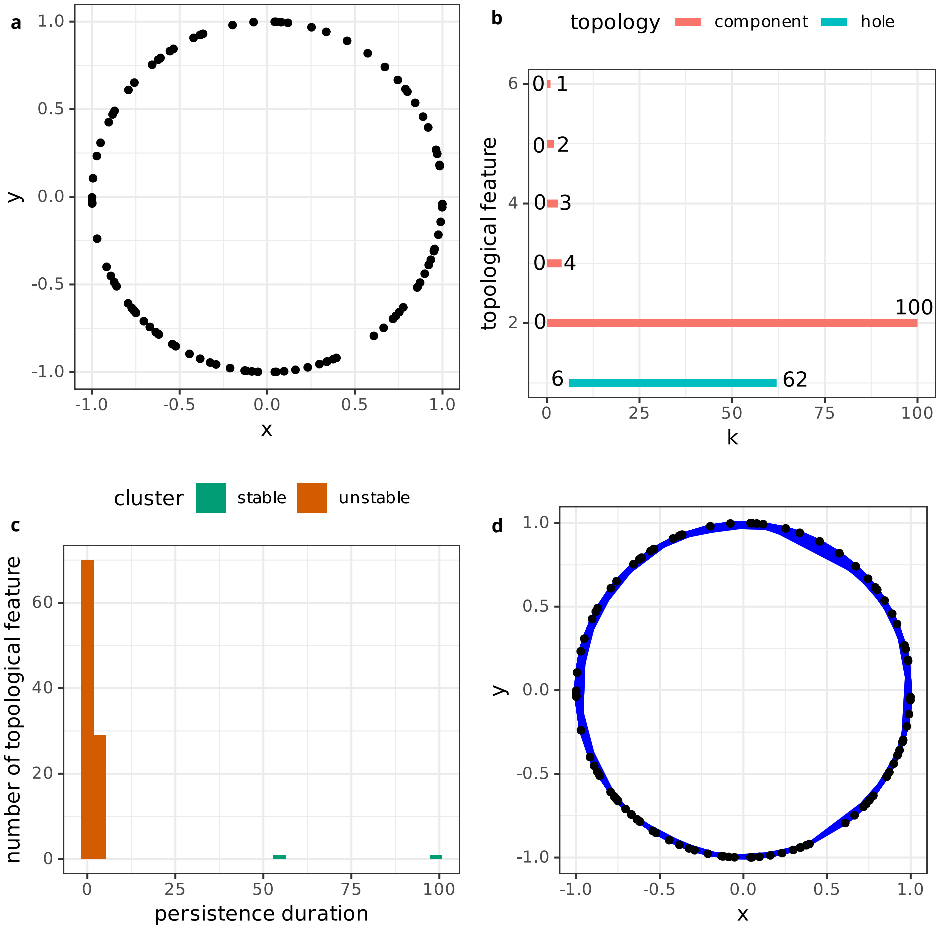

Figure S3: **a** — Sampling of 100 points on a circle **b** — Persistent diagram showing two principal topological features, a cycle (a hole) and a single component. **c** — Histogram of distribution of persistence duration of the topological features in the persistent diagram colored by k-mean clusters **d** — Datascape with $k = 6$ (birth of the hole)

References

[1] Sallaby, A. F. & Azlan, A. Analysis of Missing Value Imputation Application with K-Nearest Neighbor (K-NN) Algorithm in Dataset. *The IJICS (International Journal of Informatics and Computer Science)* **5** (2), 141 (2021). URL https://ejurnal.stmik-budidarma.ac.id/index.php/ijics/article/view/3185. https://doi.org/10.30865/ijics.v5i2.3185 .

[2] Batista, G. & Monard, M.-C. *A Study of K-Nearest Neighbour as an Imputation Method.* Vol. 30 (2002). Journal Abbreviation: Hybrid Intelligent Systems, ser Front Artificial Intelligence Applications Pages: 260 Publication Title: Hybrid Intelligent Systems, ser Front Artificial Intelligence Applications.

[3] Van Buuren, S. & Oudshoorn, K. *Flexible multivariate imputation by MICE* (Leiden: TNO, 1999).

[4] Josse, J. & Husson, F. Handling missing values in exploratory multivariate data analysis methods. *Journal de la Société Française de Statistique* **153** (2), 79–99 (2012) .

[5] Tenenbaum, J. B., Silva, V. d. & Langford, J. C. A Global Geometric Framework for Nonlinear Dimensionality Reduction. *Science* **290** (5500), 2319–2323 (2000). URL https://www.science.org/doi/10.1126/science.290.5500.2319. https://doi.org/10.1126/science.290.5500.2319, publisher: American Association for the Advancement of Science .

[6] McInnes, L., Healy, J. & Melville, J. Umap: Uniform manifold approximation and projection for dimension reduction (2020). 1802.03426.

[7] Moon, K. R. *et al.* Visualizing structure and transitions in high-dimensional biological data. *Nature Biotechnology* **37** (12), 1482–1492 (2019). URL https://www.nature.com/articles/s41587-019-0336-3. https://doi.org/10.1038/s41587-019-0336-3 .

[8] Wasserman, L. Topological data analysis. *Annual Review of Statistics and Its Application* **5**, 501–532 (2018) .

[9] Chazal, F. & Michel, B. An introduction to topological data analysis: fundamental and practical aspects for data scientists. *Frontiers in artificial intelligence* **4**, 667963 (2021) .

[10] Kerber, M. Persistent Homology – State of the art and challenges .
